# Evaluation and comparison of inflammatory and albumin-based markers in left-sided mechanical heart valve thrombosis

**DOI:** 10.34172/jcvtr.025.33245

**Published:** 2025-09-28

**Authors:** Cemalettin Yılmaz, Mustafa Ozan Gürsoy, Büşra Güvendi Şengör, Barkın Kültürsay, Semih Kalkan, Ahmet Karaduman, Ahmet Ferhat Kaya, Emrah Bayam, Mehmet Özkan

**Affiliations:** ^1^Department of Cardiology, Malazgirt State Hospital, Malazgirt, Muş, Turkey; ^2^Department of Cardiology, Kartal Kosuyolu Research and Education Hospital, Kartal, İstanbul, Turkey; ^3^Department of Cardiology, Atatürk Training and Research Hospital, İzmir Katip Çelebi University, Karabağlar, İzmir, Turkey; ^4^Department of Cardiology, Başakşehir Çam and Sakura City Hospital, Başakşehir, İstanbul, Turkey; ^5^Department of Cardiology, Muş State Hospital, Muş Center, Muş, Turkey

**Keywords:** Mechanical valve thrombosis, Obstruction, Thrombosis, Neutrophile to lymphocyte ratio, Inflammatory marker

## Abstract

**Introduction:**

Mechanical valve thrombosis (MVT), along with its associated embolic or obstructive complications, can have significant clinical implications. Studies evaluating the relationship of inflammatory and albumin-based markers such as neutrophile-lymphocyte ratio (NLR), platelet lymphocyte ratio (PLR), C-reactive protein to albumin ratio, uric acid to albumin ratio and lactate dehydrogenase to albumin ratio (LAR) with thrombosis burden in patients with left sided mechanical valves are lacking. We aimed to examine the relationship between inflammatory and albumin-based markers, and left sided MVT with and without obstruction.

**Methods:**

Eighty-eight patients (age 48.9±13.8, 72.7% female) with left-sided MVT were included in the study. Patients were divided into two groups as obstructive thrombosis (OT) and non-obstructive thrombosis (NOT).

**Results:**

NLR, PLR and LAR levels were found to be significantly higher in OT group (*P*<0.001, *P*<0.001; *P*=0.032, respectively). There was a strong correlation between OT and both NLR and PLR (r=0.736 and r=0.645). Of those markers, NLR (odds ratio (OR) 5.68, 95% confidence interval (CI) 1.96 to 16.48, *P*=0.001) was found to be the independent predictor of OT. On receiver operating characteristic (ROC) analysis, a cut-off value 2.83 of NLR (area under the curve (AUC)=0.931; 95% CI:0.877-0.984; *P*<0.001) had 86.3% sensitivity and 86.5% specificity for prediction of OT.

**Conclusion:**

Increased NLR, PLR, and LAR values were associated with OT in left-sided MVT. Of those, NLR was found to be the most sensitive and specific marker and represented as an independent predictor of OT.

## Introduction

 Mechanical valve thrombosis (MVT), along with its associated embolic or obstructive complications, can have significant clinical implications.^1^ While optimizing anticoagulation is recommended for non-obstructive thrombosis (NOT), the preferred treatment for obstructive thrombosis (OT) involves fibrinolysis or cardiac surgery.^2,3^ Therefore, it is crucial to determine the presence and type of obstruction by means of echocardiography, fluoroscopy or multislice computed tomography (MSCT). Because the diagnosis of OT becomes difficult in cases where echocardiography cannot be optimally performed, readily applicable markers that can assist in the diagnosis are needed.

 Inflammation has a triggering role in thrombus formation by stimulating the coagulation pathway.^4^ Although the relationship of thromboembolic cardiovascular diseases with current inflammatory and albumin-based markers such as neutrophile-lymphocyte ratio (NLR), platelet lymphocyte ratio (PLR), C-reactive protein (CRP) to albumin ratio (CAR) and uric acid to albumin ratio (UAR) has been reported in recent studies, there is no data on the relationship between lactate dehydrogenase (LDH) to albumin ratio (LAR) and cardiovascular diseases.^5-13^ Moreover, data evaluating the relationship of these markers with thrombosis and thrombosis burden in patients with left sided mechanical valves are lacking. The purpose of this study was to examine the relationship between current inflammatory and albumin-based markers, and left sided MVT with and without obstruction.

## Materials and Methods

**Figure 1:**
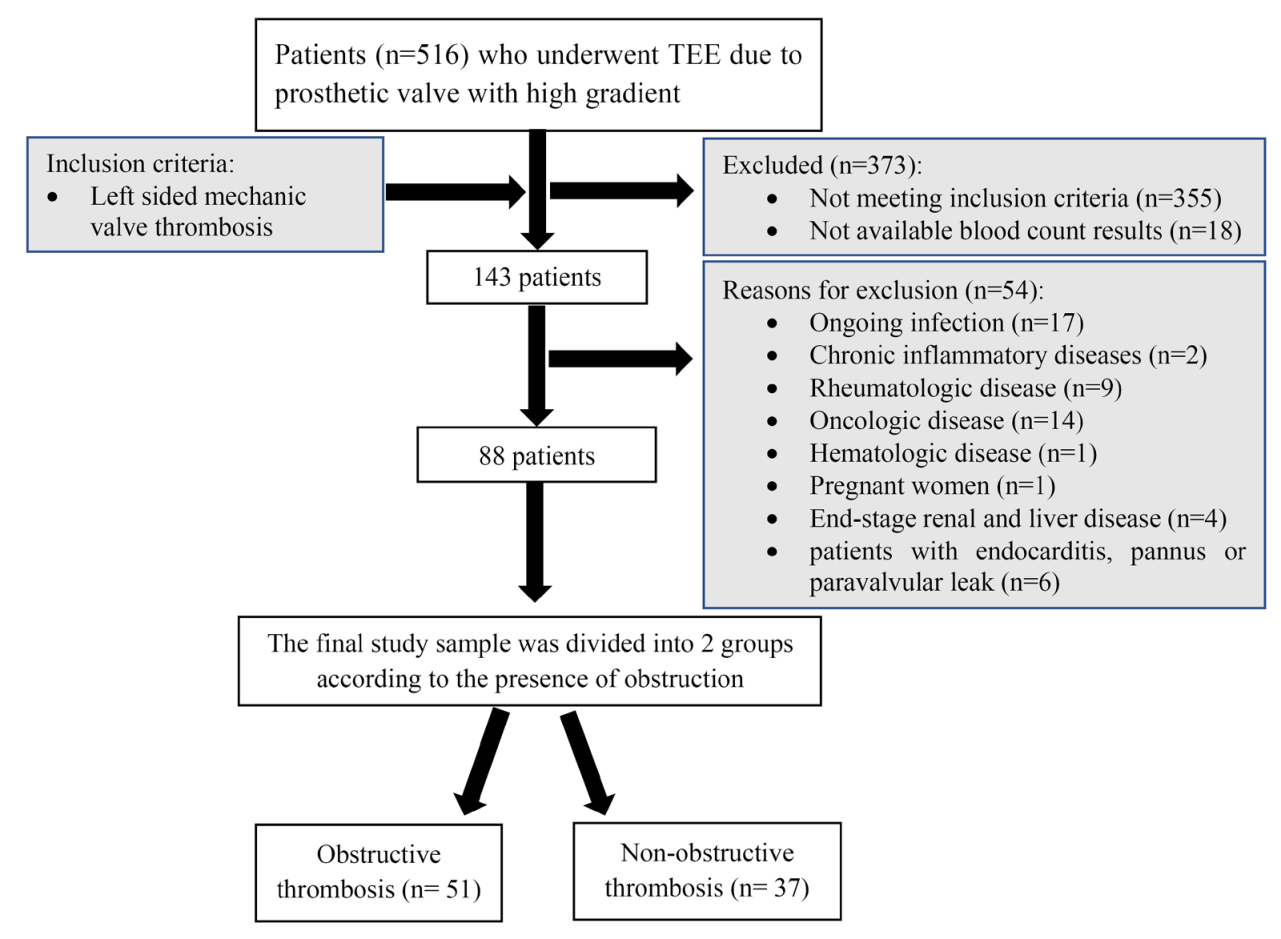
Flowchart of study population (TEE: Transesophageal echocardiography)

 The study was conducted at a tertiary care referral center in Turkey (Kartal Koşuyolu Education and Research Hospital, Kartal, Istanbul). All consecutive patients (n = 516) who underwent transesophageal echocardiography (TEE) due to prosthetic heart valve with high gradient from 2015 to 2021 were retrospectively analyzed. A total of 143 patients with left sided MVT (aortic, mitral and both) were included in the study. Patients with ongoing infection (The diagnosis of infection was based on clinical findings and patient complaints, which included symptoms such as fever, productive cough, and dysuria. Additionally, the presence of bacteria in blood, urine, or sputum cultures was used as a definitive diagnostic criterion.), chronic inflammatory diseases, and rheumatologic, oncologic, hematologic disease, pregnant women, those with end stage renal and liver disease and patients with endocarditis, pannus or paravalvular leak detected in echocardiographic evaluation were excluded from the study. Besides, patients in whom thrombus, pannus or vegetation could not be clearly differentiated were also excluded from the study. Finally, 88 patients with left sided MVT were included in the study. Patients were divided into two groups as OT and NOT. Flowchart of this study population was demonstrated in Figure 1. Ethical approval was obtained from the Ethical Board of Koşuyolu High Specialization Training and Research Hospital (Approval number: 2022/9/589, Approval date: 10/05/2022), and the study was conducted in accordance with the principles outlined in the Declaration of Helsinki. Every patient provided written informed consent prior to participating in the study.

 Demographic and clinical characteristics of the subjects and laboratory data including complete blood count and blood biochemistry were obtained from the hospital database system (FONET®, Information Technology Incorporation, Turkey) and electronic health records (e-Nabiz, Turkey). The blood values obtained from venous blood samples at the time of admission, before any medication was administered, were recorded from medical reports. The NLR, PLR, CAR, LAR, UAR were calculated using the following formulas: neutrophil count/lymphocyte count, platelet count/lymphocyte count, CRP level/albumin level, lactate dehydrogenase level/albumin level, uric acid level/albumin level, respectively, derived from peripheral blood (per mm^3^). International normalization ratio (INR) values that were < 70% of time in the therapeutic range (TTR) were indicative of inadequate anticoagulation.^14^

###  Echocardiography

 The study subjects were first evaluated with transthoracic echocardiography (TTE) using Philips EPIQ 7 (Philips Medical Systems, Andover, Massachusetts, USA) and S5-1 sector array transducer. Following TTE, two-dimensional (2D) and three-dimensional (3D) TEE studies were performed by using an X7-2t transducer on the same device. A thrombus was identified via echocardiography as a homogeneous mobile or fixed mass with echo density likely to myocardium on the mechanic valve.^1^ Distinction of thrombus from excessive pannus overgrowth or vegetation was made mainly on the basis of echocardiographic findings. Multimodal imaging, including fluoroscopy and MSCT, was used to aid in the diagnosis of MVT in selected cases. In mitral mechanical prosthetic valves, the possible and definite obstruction values for effective orifice area (EOA) and mean gradient (MG) were considered as follows:

- Possible obstruction values: EOA of 1-2 cm^2^ and MG of 6-10 mmHg.
- Definite obstruction values: EOA < 1 cm^2^ and MG ≥ 10 mmHg.

In aortic mechanical prosthetic valves, the possible and definite obstruction values for MG, doppler velocity index (DVI) and acceleration time (AT) were considered as follows:

- Possible obstruction values: The MG of 20-34 mmHg, DVI of 0.25-0.34 and AT of 80-100 ms.
- Definite obstruction values: The MG of ≥ 35 mmHg, DVI of < 0.25 and AT > 100 ms.

This study utilized the specified cut-off values from recent guidelines to determine the criteria for prosthetic valve obstruction.^15^ Presence of thrombus on TEE without obstructive (possible or definitive) hemodynamic changes on Doppler echocardiography indicated NOT.^16,17^ Based on the echocardiographic findings, patients with prosthetic MVT were divided into two groups as OT and NOT.

###  Statistical Analysis

 Statistical analysis was performed with R (Vienna, Austria) and JAMOVI v.2.3.21 software (The JAMOVI Project, Sydney, Australia). The normality of the distribution of the data was analyzed with the Kolmogorov–Smirnov test. Continuous variables are presented as medians and interquartile ranges or as mean standard deviation (SD), as appropriate. Categorical variables were expressed as number and percentages and Pearson’s chi-square or Fisher’s exact tests were used to evaluate the differences. Group comparisons for continuous variables were tested using Student’s-*t* or Mann–Whitney U whichever appropriate. Values with significant differences between the groups were evaluated with Pearson or Spearman correlation analysis according to their distribution. A receiver-operating characteristics (ROCs) curve analysis was performed to identify the optimal cut-off point of NLR, PLR, and LAR to predict obstructive MVT. Multivariable logistic regression analysis was performed to identify predictors of OT. *p* < 0.05 was accepted as statistically significant.

## Results

 The study population (n = 88, age 48.9 ± 13.8, 72.7% female) included patients with OT [51 (58%) patients; n = 6 aortic, n = 42 mitral, n = 3 both aortic and mitral] versus patients with NOT [37 (42%) patients; n = 8 aortic, n = 28 mitral, n = 1 both aortic and mitral]. After using 2D and 3D TEE as the primary imaging modality for diagnosing aortic MVT (n = 18), additional imaging tools, including fluoroscopy (in 6 patients, 33.3%) and MSCT (in 1 patient, 5.5%), were required to support the diagnosis in 6 patients (33.3%). Thebaseline clinical and demographic characteristics of the whole population are shown in Table 1. The mean ages of the population in OT and NOT groups were 50.5 ± 13.5, 46.7 ± 14.1 years, respectively (*P* = 0.211). The comparison of body mass index (BMI) between OT and NOT groups showed similar results, with mean BMI values of 24.4 ± 7.1 and 23.9 ± 7.7 kg/m^2^, respectively (*P* = 0.74). The most prevalent valve genus was St. Jude Medical bileaflet valve (St. Jude Medical Inc., St. Paul, Minnesota). There were no differences between the groups in terms of gender, history of hypertension (HT), diabetes mellitus (DM), smoking, coronary artery disease (CAD), cerebrovascular disease, and acetylsalicylic acid (ASA) use. While ejection fraction (EF) was comparable in both groups (OT *vs.* NOT, 54.9 ± 9.8 and 55.3 ± 11, respectively), atrial fibrillation (AF) prevalence and the New York Heart Association (NYHA) functional class was significantly higher in OT group compared to NOT group (*P* < 0.001, both). In addition, the mean thrombus area (cm^2^) was 1.31 ± 0.52 cm^2^ in the OT group and 0.85 ± 0.56 cm^2^ in the NOT group (*P* = 0.046).

**Table 1:** Clinical characteristics of the study patients according to the presence of obstruction

**Parameters**	**OT** **n=51 (58%)**	**NOT** **n=37 (42.0%)**	***P*** **-value**
Age (years), mean ± SD	50.5 ± 13.5	46.7 ± 14.1	0.211
Gender, n (%)			
Male	13 (25.4%)	11 (29.7%)	0.664
Female	38 (64.7%)	26 (70.2%)	
Body mass index (kg/m^2^)	24.4 ± 7.1	23.9 ± 7.7	0.74
HT, n (%)	15 (29.4%)	9 (24.3%)	0.602
DM, n (%)	15 (29.4%)	7 (18.9%)	0.256
Smoking, n (%)	3 (5.8%)	3 (8.1%)	0.687
CAD, n (%)	5 (9.8%)	6 (16.2%)	0.375
EF %, mean ± SD	54.9 ± 9.8	55.3 ± 11	0.869
Mean thrombus area (cm^2^)	1.31 ± 0.52	0.85 ± 0.56	**0.046**
Atrial Fibrillation, n (%)	36 (70.5%)	12 (32.4%)	**<0.001**
Cerebrovascular Disease, n (%)	10 (19.6%)	8 (21.6%)	0.82
NYHA Classification, n (%)			
NYHA 1	0 (0%)	8 (21.6%)	**<0.001**
NYHA 2	12 (23.5%)	16 (43.2%)	
NYHA 3	37 (72.5%)	13 (35.1%)	
NYHA 4	2 (3.9%)	0 (0%)	
Patient Taking ASA, n (%)	6 (11.7)	3 (8.1)	0.581

ASA, acetylsalicylic acid; OT, obstructive thrombosis; NOT, nonobstructive thrombosis; HT, hypertension; DM, diabetes mellitus; CAD, coronary artery disease; EF, ejection fraction; NYHA, New York Heart Association; SD, standard deviation.

**Figure 2:**
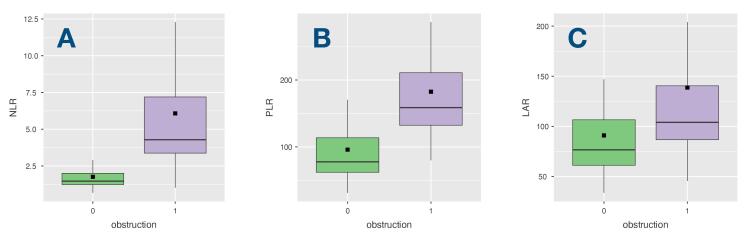
Box plots showing the relationship between presence of obstruction and **(A)** NLR, **(B)** PLR and **(C)** LAR in patients with mechanical heart valve thrombosis

 The comparison of laboratory parameters according to the presence of obstruction is shown in Table 2. White blood cell (WBC) counts, platelet counts, neutrophil counts, urea and LDH levels were significantly higher, and lymphocyte counts were significantly lower in OT group compared to NOT group (11.1 ± 3.7 *vs*. 8.7 ± 2.6, *P* = 0.01; 284 ± 95.9 *vs.* 222.4 ± 78.5, *P* = 0.02; 8.3 ± 3.4 *vs.* 3.5 ± 1.2, *P* < 0.001; 46.9 ± 27.4 *vs.* 32.4 ± 18.7, *P* = 0.004; 515.7 ± 453 *vs.* 344.9 ± 144.1, *P* = 0.014; 1.7 ± 0.7 *vs.* 2.6 ± 0.8, *P* < 0.001; respectively). There were no differences between the groups in terms of international normalized ratio (INR), D-Dimer levels, hemoglobin (HB), hematocrit (HCT), monocyte counts, parameters of cholesterol, creatinine, glomerular filtration ratio (GFR), aspartate aminotransferase (AST), alanine aminotransferase (ALT), albumin levels, CRP, and uric acid. However, number of patients with inadequate anticoagulation (time in therapeutic range (TTR) < 70%) was higher in OT group (*P* < 0.001). NLR, PLR and LAR levels which were derived from these blood parameters were found to be significantly higher in OT group (6.0 ± 5.6 *vs.* 1.7 ± 0.7, *P* < 0.001; 182.4 ± 89.0 *vs.* 95.9 ± 63.3, *P* < 0.001; 138.6 ± 126.5 *vs.* 90.9 ± 49.5, *P* = 0.032; respectively). In addition, Figure 2 displays box plots illustrating the association between presence of obstruction and (A) NLR, (B) PLR, and (C) LAR in patients diagnosed with MVT. CAR and UAR levels were found to be similar between the groups.

**Table 2:** Comparison of laboratory parameters according to the presence of obstruction

**Parameters**	**OT** **n=51 (58%)**	**NOT** **n=37 (42.0%)**	***P*** **-value**
Glucose (mg/dl)	129.8 ± 7	127.2 ± 101.7	0.887
INR	2.5 ± 1.5	2.1 ± 0.9	0.244
TTR < 70%, n (%)	34 (66.7)	10 (27)	< 0.001
D-Dimer (mg/L)	2.3 (1.6-5.5)	1.1 (0.8-2.7)	0.098
WBC (10^3^/μL)	11.1 ± 3.7	8.7 ± 2.6	**0.01**
HB (g/dL)	11.5 ± 1.9	12.2 ± 2.1	0.123
HCT (%)	35.5 ± 6.2	37.9 ± 5.2	0.056
MCV (fL)	82.5 ± 8.6	83.6 ± 7.6	0.572
RDW (%)	17 ± 3.3	16.3 ± 2.5	0.254
Platelet (10^3^/μL)	284 ± 95.9	222.4 ± 78.5	**0.02**
MPV (fL)	8.9 ± 1.2	10.6 ± 13.7	0.382
Neutrophil (10^3^/μL)	8.3 ± 3.4	3.5 ± 1.2	**<0.001**
Lymphocyte (10^3^/μL)	1.7 ± 0.7	2.6 ± 0.8	**<0.001**
Monocyte (10^3^/μL)	0.7 ± 0.3	0.6 ± 0.2	0.058
Total Cholesterol (mg/dL)	189.9 ± 50.8	186.3 ± 43.8	0.745
Triglyceride (mg/dL)	147.1 ± 89.8	155.5 ± 71.3	0.669
HDL-C (mg/dL)	40.6 ± 11.6	42.9 ± 10.7	0.378
LDL-C (mg/dL)	128.7 ± 53.4	112.4 ± 36	0.145
GFR (ml/min/1.73 m^2^)	85.1 ± 32.1	96.3 ± 27.4	0.091
Creatinine (mg/dL)	1.0 ± 0.4	0.85 ± 0.35	0.093
Urea (mg/dL)	46.9 ± 27.4	32.4 ± 18.7	**0.004**
ALT (U/L)	23.6 ± 13.7	32.8 ± 60.3	0.296
AST(U/L)	31.5 ± 14.6	28.8 ± 13.8	0.393
Albumin(g/dL)	3.8 ± 0.5	3.9 ± 0.5	0.254
CRP (mg/dL)	6.4 ± 5.8	5.2 ± 3.9	0.253
LDH (U/L)	515.7 ± 453	344.9 ± 144.1	**0.014**
Uric acid (mg/ dL)	6.7 ± 2.2	6.1 ± 1.7	0.159
NLR	6.0 ± 5.6	1.7 ± 0.7	**<0.001**
PLR	182.4 ± 89.0	95.9 ± 63.3	**<0.001**
CAR	1.7 ± 1.7	1.4 ± 1.3	0.3
LAR	138.6 ± 126.5	90.9 ± 49.5	**0.032**
UAR	1.8 ± 0.5	1.5 ± 0.5	0.072

ALT, alanine transaminase; AST, aspartate aminotransferase; CRP; C reactive protein; CAR; GFR, glomerular filtration rate; HCT, hematocrit; HDL-C, high-density lipoprotein cholesterol; INR, international normalized ratio; LDH, lactate dehydrogenase; LDL-C, low-density lipoprotein; MCV, mean corpuscular volume; MPV, mean platelet volume; NOT, non-obstructive thrombosis; OT, obstructive thrombosis; RDW, red cell distribution width; WBC, white blood cell; CAR, CRP to albumin ratio; LAR, lactate dehydrogenase to albumin ratio; NLR, neutrophile to lymphocyte ratio; NOT, non-obstructive thrombosis; OT, obstructive thrombosis; PLR, platelet to lymphocyte ratio; UAR, uric acid to albumin ratio.

**Figure 3:**
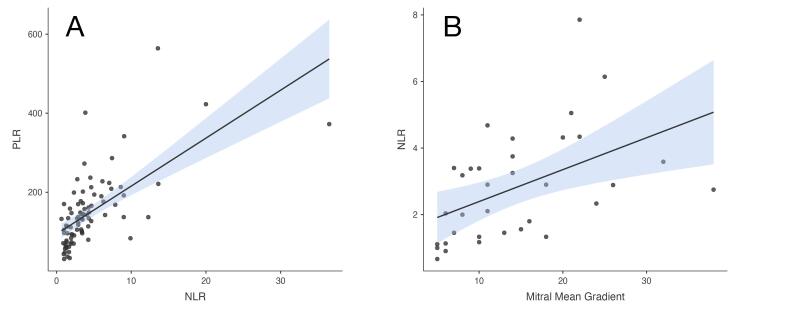
**(A)** Scatterplot of NLR-PLR correlation and **(B)** Scatterplot of NLR-mitral MG correlation

 There was a strong correlation between OT and both NLR and PLR (r = 0.736 and r = 0.645, respectively), while there was a moderate correlation between OT and LAR (r = 0.353). In Figure 3, (A) scatterplot of NLR-PLR correlation and (B) scatterplot of NLR-mitral MG correlation were shown.

**Figure 4:**
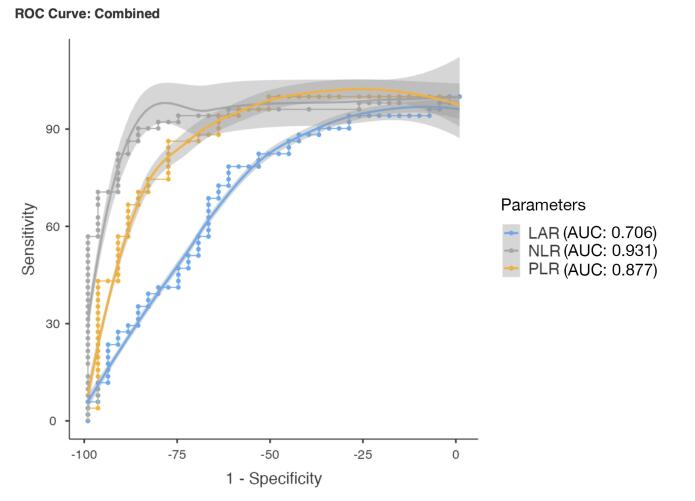
Receiver-operating characteristic curve analysis to identify the optimal cut-off point of NLR, PLR, and LAR to predict prosthetic valve thrombosis

 ROC analysis revealed the optimal cut-off values for NLR, PLR, and LAR in predicting left-sided obstructive MVT. A cut-off value of 2.83 for NLR (area under the curve (AUC) = 0.931) demonstrated 86.3% sensitivity and 86.5% specificity, while a cut-off value of 125.5 for PLR (AUC = 0.877) exhibited 78.4% sensitivity and 78.4% specificity. Additionally, a cut-off value of 84.71 for LAR (AUC = 0.706) showed 78.4% sensitivity and 62.16% specificity (Figure 4, Table 3).

**Table 3:** Assessment of inflammatory parameters for predicting obstructive thrombosis: Sensitivity, specificity, predictive values, and AUC

**Inflammatory parameters**	**Sensitivity (%)**	**Specificity (%)**	**PPV (%)**	**NPV (%)**	**AUC**
NLR	86.3	86.5	90.2	86.49	0.931
PLR	78.4	78.4	84.62	80.56	0.877
LAR	78.4	62.16	74.07	67.65	0.706

CAR, CRP to albumin ratio; LAR, lactate dehydrogenase to albumin ratio; NLR, neutrophile to lymphocyte ratio; NOT, non-obstructive thrombosis; OT, obstructive thrombosis; PLR, platelet to lymphocyte ratio; UAR, uric acid to albumin ratio; PPV, positive predictive value; NPV, negative predictive value; AUC, area under the curve.

 Multivariable logistic regression analysis was used to determine the independent predictors of OT. The variables that were found to be significant in the univariate analysis (PLR, NLR, LAR, AF, NYHA Class) were included in the multivariable analysis. Of those, NLR (odds ratio (OR) 5.68, 95% confidence interval (CI) 1.96 to 16.48, *P* = 0.001), and NYHA Class (OR 7.09, 95% CI 1.36 to 36.8, *P* = 0.02) were found to be the independent predictors of OT (Table 4). AUC and pseudo-R^2^ of the model are 0.957 and 0.772, respectively.

**Table 4:** Multivariable regression analysis: predictors of obstructive thrombosis (OT)

	**Coef.**	***P***	**Odds Ratio**	**95% Confidence Interval**	
				**Lower**	**Upper**
Intercept	-10.170	< 0.001	3.83e-5	1.59e-7	0.009
PLR	-0.006	0.461	0.994	0.977	1.010
NLR	1.737	0.001	5.681	1.958	16.484
LAR	0.006	0.420	1.007	0.991	1.022
NYHA Class	1.958	0.02	7.089	1.364	36.831
AF	1.710	0.057	5.534	0.953	32.131

PLR, platelet to lymphocyte ratio; NLR, neutrophile to lymphocyte ratio; LAR, lactate dehydrogenase to albumin ratio; NYHA, New York Heart Association; AF, atrial fibrillation.

## Discussion

 Three main messages are obtained from the results of our study. Firstly, in the OT group, the levels of NLR, PLR, and LAR were notably elevated in comparison to the NOT group. Furthermore, the results of this study demonstrate a robust correlation between OT resulting in hemodynamic adverse effect and increased NLR, PLR. Lastly, among the existing inflammatory and albumin-based parameters, NLR demonstrates the highest sensitivity and specificity, and serves as an independent predictor of OT. These data suggest that NLR may be the best among the accepted parameters reflecting the thrombo-inflammatory system, which has a triggering and aggravating effect on the prothrombotic process.

 In recent publications, various studies have explored the association between thrombotic cardiovascular diseases such as stent thrombosis, left ventricular thrombosis after acute myocardial infarction (AMI), deep venous thrombosis (DVT) and pulmonary thrombo-embolism (PTE), left ventricular assist device (LVAD) thrombosis, left atrial thrombosis, ischemic stroke, acute lower extremity ischemia and current inflammatory and albumin-based parameters.^5,9,11,12,18-21^

 Increased neutrophil count in response to the inflammatory response and decreased lymphocyte count due to increased stress-induced cortisol make NLR a valuable parameter.^22-24^ In fact, NLR surpasses neutrophil count as a more robust inflammatory marker.^25^ Ayça et al^5^ previously investigated the association between NLR values and stent thrombosis. Furthermore, a meta-analysis indicated a significant association between NLR and both hospitalization rate and long-term prognosis in patients with ST-elevated myocardial infarction (STEMI) who underwent percutaneous coronary intervention.^26^ In a separate study, Sia et al^21^ observed that patients with elevated NLR and PLR values had a lower rate of left ventricular thrombus resolution. Moreover, in the same study, NLR and PLR were identified as distinct predictors of thrombus resolution in cases of AMI without percutaneous coronary intervention.^21^ Kuplay et al^13^ found significantly higher NLR and PLR rates in patients with more proximal DVT, which is the third leading vascular problem worldwide. Moreover, they emphasized that NLR values are associated with thrombus burden in patients with DVT, unlike PLR values.^13^ In yet another study, NLR was found to be linked to the prognosis of patients with PTE.^27^ Ferrera et al^19^ reported that NLR measured at 4-6 months in patients with LVAD implantation, was associated with increased mortality and major adverse events such as infection, stroke and pump thrombosis. In a study involving patients with non-valvular AF, Yalcin et al^11^ showed the relationship between increased NLR value and the presence of left atrial thrombus, which precedes a stroke. In recently published study of Deng et al,^10^ NLR value was found to be an independent predictor for left atrial thrombus and spontaneous echo contrast in patients with non-valvular AF. Additionally, Suh et al^9^ revealed that elevated NLR levels serve as an independent risk factor for ischemic stroke in healthy adults. However, studies examining the relationship between valve diseases and NLR are insufficient in the literature. A study involving 62 patients with mitral stenosis noted that NLR value was significantly higher in the spontaneous echo contrast positive group.^28^ Gürsoy et al^29^ reported that NLR value was significantly higher in patients with OT compared to NOT in a subgroup analysis of their study in patients with mitral valve prosthesis. This relationship was explained by acute hemodynamic changes in the left atrium. Likewise, in our study, NLR was notably higher in the group with OT. Since our study population included left-sided mechanical valves, it is insufficient to explain this response by changes occurring in the left atrium alone. These findings may suggest that pressure changes in both the left atrium and the left ventricle due to thrombosis stimulate the inflammatory response.

 In our study, the NLR cutoff value of 2.83 was determined through ROC curve analysis to assess its predictive value for OT. Although this threshold may appear isolated at first glance, it is consistent with findings from previous studies in various thrombotic conditions. For instance, an NLR cutoff of > 2.56 was reported in patients with proximal DVT.^13^ Similarly, Yalçın et al^11^ identified a cutoff of 2.59 for predicting left atrial thrombus in patients with nonvalvular AF. Another study evaluating left atrial thrombus and spontaneous echo contrast found a cutoff of 2.57.^10^ Additionally, in patients with mitral stenosis, a slightly higher cutoff of 3.1 was shown to predict spontaneous echo contrast with 80% sensitivity and 72% specificity.^28^ These data suggest that the NLR threshold observed in our study is within a clinically relevant range and supports its utility as a marker in thrombotic risk assessment.

 Stimulation of megakaryocyte cells in response to inflammation causes an increase in platelet count, contributing to thrombotic cardiovascular diseases.^30,31^ Increase in platelet counts and decrease in lymphocyte counts make PLR an important parameter for assessing the inflammatory process. A study in patients with malignancy demonstrated that increased PLR values were associated with an increased risk of DVT.^12^ In another study that compared patients with mitral mechanical valve to the control group (consisting of individuals with normal functional mitral mechanical valve), it was observed that the NLR and PLR were higher in the group with MVT.^29^ Moreover, PLR was found to be an independent predictor for MVT.^29^ However, in the subgroup analysis of this study, unlike our study, PLR values were similar in the OT and NOT groups. The discrepancy between our study and the previous study could be attributed to differences in patient cohorts. We believe that our study is more comprehensive and widely applicable since it included patients with left-sided MVT, unlike the previous study that focused solely on mitral valve thrombosis.

 Duman et al^8^ showed that increased CRP, a traditional inflammatory marker, and decreased albumin levels were associated with thrombus burden in patients with AMI. They also showed that the CAR ratio was an independent predictor of thrombus burden in the same study.^8^ In the subgroup analysis of the study by Gürsoy et al,^29^ CRP level was notably higher in the group with OT compared to the group NOT. Interestingly, in our study, CRP, albumin, and CAR, all of which have previously been linked to thrombus burden, did not show significant differences between the groups.

 LDH is an intracellular enzyme involved in cell metabolism and turnover, and it is present in various cell types, including erythrocytes and cardiac cells.^32^ In a recent study, elevated LDH levels in lymphoma patients were identified as a prognostic indicator for the development of DVT.^18^ Previous studies have demonstrated that LDH released from hemolyzed erythrocytes under heightened shear stress can be utilized as a diagnostic marker for LVAD thrombosis.^32,33^ LAR, previously associated with prognosis in colorectal cancers and lower respiratory tract infections, has not been adequately studied in thrombotic cardiovascular disease.^34,35^ LDH levels and a new parameter, LAR, were significantly higher in patients with OT in our study group. Furthermore, moderate correlation was observed between LAR and OT.

 UAR, a marker recently associated with mortality in patients with STEMI, was also found to be linked with coronary no-reflow in another study.^6,7^ In our study, on the other hand, UAR was found to be similar in both groups. Additional comprehensive studies are required to assess the association between UAR and prosthetic valve diseases, whose relationship with cardiovascular diseases has just been clarified.

 Low EF and presence of AF are associated with MVT.^1^ In contrast to the prevailing literature, our study did not reveal any significant relationship between OT and EF. This result can be explained by the small patient population and we do not think this result is generalizable. Similar to the literature, the incidence of AF was remarkably high in patients with OT. AF is a well-known thrombosis risk factor, particularly in the context of stroke and systemic embolism, due to irregular and ineffective atrial contractions that cause blood flow stasis and lead to clot formation.^36^ Moreover, the relationship between AF and MVT has been demonstrated in previous studies.^14^ In our study, the multivariable analysis revealed a borderline significant relationship between AF and OT in patients with MVT, suggesting a potential link between them. This relationship may indicate that AF not only induces thrombosis but also exacerbates thrombus formation by disrupting the mechanical function of the left atrium. However, this association has not been statistically validated, and further investigation is needed. Furthermore, inadequate anticoagulation is one of the most significant causes of MVT. Our results revealed that the rate of inadequate anticoagulation was notably higher in the OT group compared to NOT group. This finding highlights the importance of proper anticoagulation management in patients with mechanical heart valves to prevent the occurrence of thrombotic complications. Consequently, we suggest to improve the potential strategies for improving anticoagulation therapy to reduce the risk of MVT in this patient population. In addition, mean thrombus area (cm^2^) was significantly higher in OT group over the NOT group. One factor contributing to prosthetic MVT is not only the thrombus size but also its location, which can disrupt valve movement. The mechanical impact of a thrombus attached to the valve hinge differs from that of a thrombus attached to the annulus. Obstruction is influenced by both the number and size of thrombi, as well as their specific locations. Therefore, it is essential to consider that even a small thrombus can lead to obstruction. However, it is important to acknowledge that our study solely focused on assessing thrombus area. Consequently, the lack of sufficient data regarding the localization and number of thrombi in the study population can be considered as a limitation of our research. Future investigations should aim to address these aspects to provide a more comprehensive understanding of the thrombotic process in the context of our study.

 Although NLR, PLR and LAR values are significantly associated with obstruction, NLR stands out as an independent predictor of OT with remarkable sensitivity and specificity, making it more significant and valuable compared to other markers. In fact, high NLR values may indicate a more aggressive progression of thrombosis. Therefore, we can consider NLR as the parameter that most accurately reflects the presence of hemodynamic changes in MVT. The findings of this study may serve as a basis for future larger prospective studies, guiding decision-making implications regarding the significance of high NLR values in patients with left sided MVT.

 Several limitations are present in this study. This study was a single-center retrospective analysis that focused on patients with left-sided MVT. Inflammatory markers such as interleukin-1,6 or tumor necrosis factor alpha (TNFα) were not evaluated in this study. Analyzing of these markers could have strengthened the study. However, it is not feasible to utilize these markers in routine clinical practice. In addition, the definitive diagnosis of thrombus has not been confirmed histopathologically. Moreover, we calculated these parameters only at referral rather than the evaluating temporal trend over the follow-up period. Therefore, the impact of biomarkers on mortality and other follow-up clinical outcomes was not evaluated.

## Conclusion

 Increased NLR, PLR, and LAR values were significantly associated with OT in patients with left-sided MVT. Of those, NLR was found to be the most sensitive and specific marker and represented as an independent predictor of mechanical valve obstruction. We can affirm NLR as the parameter that best reflects the presence of hemodynamic changes in left sided prosthetic heart valve thrombus.

## Competing Interests

 The authors declare that they have no conflict of interest.

## Ethical Approval

 Ethical approval was obtained from the Ethical Board of Koşuyolu High Specialization Training and Research Hospital (Approval number: 2022/9/589, Approval date: 10/05/2022).
